# Social determinants of sex differences in disability among older adults: a multi-country decomposition analysis using the World Health Survey

**DOI:** 10.1186/1475-9276-11-52

**Published:** 2012-09-08

**Authors:** Ahmad Reza Hosseinpoor, Jennifer Stewart Williams, Ben Jann, Paul Kowal, Alana Officer, Aleksandra Posarac, Somnath Chatterji

**Affiliations:** 1 Department of Health Statistics and Information Systems, World Health Organization, 20 Avenue Appia, Geneva, 27 1211, Switzerland; 2 Research Centre for Gender Health & Ageing, Faculty of Health, University of Newcastle, Callaghan, 2308, Australia; 3 Institute of Sociology, University of Bern, Lerchenweg 36, 3000, Bern 9, Switzerland; 4 Department of Violence, Injury Prevention and Disability, World Health Organization, 20 Avenue Appia, 1211, Geneva 27, Switzerland; 5 Social Protection and Labor, Human Development Network, The World Bank, Washington, DC, 20433, USA

**Keywords:** Disability, Chronic disease, Functional limitation, Ageing, Aging, Equity, Inequalities, Social determinants

## Abstract

**Introduction:**

Women represent a growing proportion of older people and experience increasing disability in their longer lives. Using a universally agreed definition of disability based on the International Classification of Functioning, Disability and Health, this paper examines how, apart from age, social and economic factors contribute to disability differences between older men and women.

**Methods:**

World Health Survey data were analyzed from 57 countries drawn from all income groups defined by the World Bank. The final sample comprises 63638 respondents aged 50 and older (28568 males and 35070 females). Item Response Theory was applied to derive a measure of disability which ensured cross country comparability. Individuals with scores at or above a threshold score were those who experienced significant difficulty in their everyday lives, irrespective of the underlying etiology. The population was then divided into “disabled” vs. “not disabled”. We firstly computed disability prevalence for males and females by socio-demographic factors, secondly used multiple logistic regression to estimate the adjusted effects of each social determinant on disability for males and females, and thirdly used a variant of the Blinder-Oaxaca decomposition technique to partition the measured inequality in disability between males and females into the “explained” part that arises because of differences between males and females in terms of age and social and economic characteristics, and an “unexplained” part attributed to the differential effects of these characteristics.

**Results:**

Prevalence of disability among women compared with men aged 50+ years was 40.1% vs. 23.8%. Lower levels of education and economic status are associated with disability in women and men. Approximately 45% of the sex inequality in disability can be attributed to differences in the *distribution* of socio-demographic factors. Approximately 55% of the inequality results from differences in the *effects* of the determinants.

**Conclusions:**

There is an urgent need for data and methodologies that can identify how social, biological and other factors separately contribute to the health decrements facing men and women as they age. This study highlights the need for action to address social structures and institutional practices that impact unfairly on the health of older men and women.

## Introduction

Women represent a growing proportion of all older people, but added survival means increasing disability associated with chronic conditions such as cardiovascular and respiratory disease, cancer and diabetes, and functional limitations that impact on daily living [1-4]. What is not well understood is the extent to which, apart from age, social and economic factors differently contribute to disability in older men and women. This paper analyses differences in disability prevalence between men and women aged 50 and over, using multi-country data from the World Health Survey (WHS).

Disability spans physical, mental and psychosocial functioning, encompassing a complex suite of conditions, activities and relationships [2,5]. This study adopts the most recent definition of disability based on the International Classification of Functioning, Disability and Health (ICF) developed by the World Health Organization (WHO) [6]. Under the ICF “disability” includes limitations in functioning resulting from interactions between the individual’s “health condition” (e.g. diseases, injuries, and disorders) and “environmental factors”. These decrements in body function and capacity are not necessarily linked to a particular health condition [5].

At a population level, “social determinants” or the conditions in which people are born, live, work and age, influence morbidity and mortality [7]. Differences in health between men and women are attributed to both biological and social factors [8-12]. Social determinants contribute to gender inequalities and inequities – unfair and avoidable differences in health [11]. In many countries age adjusted disability prevalence rates are higher for women and those in the poorest wealth quintile [3].

A study of gender differences in health at ages 50 and older in 11 European countries, England and the United States showed that, after adjusting for age, women were more likely than men to have disabling, non-lethal conditions including functioning problems and depressive symptoms [13]. Socioeconomic disadvantage, measured by occupation, lower levels of income and education in higher-income countries is associated with greater disability among older women [14-16]. Evidence from studies in lower- and middle-income countries also shows association between social factors and disability in older women [10,11,17-20].

The aims of this study are to: identify and assess how disability in males and females aged 50 and older is separately associated with a range of social factors; measure and evaluate the effects of sex (i.e. being male or female) on disability in people aged 50 and older after adjusting for the effects of age and the social determinants, and decompose the extent to which these different factors explain male–female differences in disability in older adults.

This study fills several gaps in the existing literature with regard to the determinants of disability in older adult populations for several reasons. Firstly the data derive from a large multi-country survey that used the same instrument and methodology to collect health and other information at the individual level across high-, middle- and low-income countries. Secondly, using an internationally agreed definition of disability based on the ICF, an advanced statistical method is applied to create a measure of disability which ensured cross country comparability. Thirdly, the study focusses specifically on the social determinants of health in men and women [7]. Finally, we apply a decomposition method to show how age and social factors contribute to the disability difference between men and women in this large international dataset.

## Methods

### Sample and data collection

The WHS was conducted by the WHO in 2002–2004 to provide representative and comparable population data on the health status of adults, aged 18 years and older, in 70 countries from all regions of the world [21] (see http://www.who.int/healthinfo/survey/en/index.html). All country samples were probabilistically selected. In China, Comoros, the Republic of the Congo, Côte d'Ivoire, India, and the Russian Federation, the WHS was carried out in geographically limited regions. To adjust for the population distribution represented by the United Nations Statistical Division (http://unstats.un.org/unsd/default.htm) and also non-response, post-stratification corrections were made to the sampling weights [22,23].

Our study includes 57 countries drawn from all income groups as defined by the World Bank [24]. The remaining 13 countries in the WHS were excluded here because either information on sampling weights was not available (11 countries) or there were insufficient data to create the household wealth index, which is one of our independent variables and a measure of socioeconomic status. The final sample comprises 63638 respondents aged 50 and older. Two pooled datasets of 28568 males and 35070 females are analyzed. (Additional file 1: Table S1 shows each country’s final sample by sex.)

### Dependent variable

For the purposes of our study “disability” is defined as decrement in functioning beyond a specific threshold. The construct is based on 16 questions in the WHS, grouped into eight health and functioning domains: vision, mobility, self-care, cognition, interpersonal activities, pain and discomfort, sleep and energy, and affect [25,26]. Self-reported response categories to these questions were: no difficulty, mild difficulty, moderate difficulty, severe difficulty, and extreme difficulty. See Additional file 2: Table S2 for list of WHS questions.

An Item Response Theory (IRT) partial credit model [27] was used to construct a composite measure of disability for each individual. The measure, which was previously developed for the WHO Report on Disability [3], allows a parsimonious measurement of the disability construct using a constellation of items from the eight domains of health in the WHS. The scale ranged from 0 (=no difficulty) to 100 (=complete difficulty).

The average of scores from respondents who reported extreme difficulties or total inability in any of the eight domains and who reported having been diagnosed with a chronic disease – such as arthritis, angina, asthma, diabetes, and depression – was around 40. A cut-point of 40 was used to divide the population into “disabled” vs. “not disabled”. Scores at or above this threshold identify those individuals experiencing significant difficulty in their everyday lives, irrespective of the underlying etiology [3]. This difficulty may be due to a range of health conditions such as arthritis, angina, alcohol dependence, depression or low vision.

### Independent variables

In addition to sex, the independent variables (all categorical) are: participants' age (using categories 50–54, 55–59, 60–64, 65–69, 70–74, 75–79, and 80+ years); marital status (married/cohabiting, never married, divorced/separated/widowed); educational level (no education/incomplete primary, complete primary, secondary/high school, college completed or above); employment status (currently in paid employment, not working for pay); area of residence (rural, urban), and country of residence. The selection of these covariates was guided by the findings of the Commission on Social Determinants [7,28].

A dichotomous hierarchical ordered probit model was used to develop an index of household economic status based on owning selected assets. The premise is that wealthier households are more likely to own a given set of assets. Asset-based approaches avoid some of the reporting biases that arise from using self-reported income. The effects of asset ownership and household characteristics on household wealth were simultaneously estimated with the hierarchical error term at the household level. The model produced asset cut points representing the threshold on the wealth scale above which a household is more likely to own a particular asset. This “asset ladder” was then applied to every household in each survey to produce adjusted estimates of household wealth. The asset-based index [29] has previously been used in equity-analyses in low- and middle-income countries [30], in analyses of the WHS, and in other studies [31,32]. The index was divided into wealth quintiles within each country.

### Analysis

We first computed the overall prevalence of disability for males and females, and the prevalence of disability by each socio-demographic factor. We then used multiple logistic regression to estimate the adjusted effects of each social determinant on disability by sex. Finally, we used a variant of the so called Blinder-Oaxaca decomposition technique [33] to partition the measured inequality in disability between males and females into two components. This first component is the “explained” part that arises because the two groups, on average, have different values for the known characteristics (i.e. the characteristics that were used as determinants in the underlying logistic regression model). The second component is the “unexplained” part which is attributed to the differential effects that the characteristics have on each group as well as other factors not included in the logistic regression model [34].

Decomposition results were computed employing the “Oaxaca” command for Stata [35] with the “pooled” and the “logit” options specified. The “pooled” option uses the coefficients from a pooled model over both groups (including a group indicator) as the reference coefficients [36]. The “logit” option causes a nonlinear decomposition based on logistic regression to be computed, as proposed by Yun [37]. Sampling weights that take into account the selection probability of the individual were included in the analysis. These weights reflect each country's population, in such a way that if the sample size for two given countries are the same (but the population sizes of the countries are different), more weight is given to the country with a larger population when calculating the pooled estimates. Allowance was made for the non-independence of observations within each survey cluster. All analyses were carried out using Stata 11 (StataCorp, 2009).

## Results

Table 1 shows that prevalence of disability among women aged 50 years and older was nearly double that of men (40.1% vs. 23.8%). Table 1 also shows the sex-specific prevalence of disability by different levels of the independent variables. Disability prevalence increases with advancing age. Both for men and women, married/cohabiting individuals are less prone to be disabled than those who are divorced/separated/widowed. Disability is negatively associated with higher household economic status and higher educational levels for both men and women. Furthermore, disability is less prevalent among those in paid employment and among respondents living in urban areas.

**Table 1:** Prevalence of disability in men and women aged 50 and older by determinants

	**Men**		**Women**	
	Estimate*	**SE**	Estimate*	**SE**
**Overall**	23.8	0.6	40.1	0.7
**Age**				
50-54 years	15.0	0.9	27.3	1.1
55-59 years	15.9	1.0	30.5	1.3
60-64 years	21.8	1.2	41.9	1.6
65-69 years	31.9	1.6	44.7	2.3
70-74 years	36.0	2.0	53.5	1.8
75-79 years	44.5	2.4	56.3	1.9
80+ years	45.5	2.6	68.9	2.0
**Marital status**				
Married/cohabiting	22.7	0.6	33.9	0.9
Never married	21.9	3.1	34.6	2.4
Divorced/separated/widowed	33.2	1.7	49.4	1.1
**Education**				
No education	31.9	1.2	47.2	1.3
Incomplete primary	30.9	1.5	44.6	1.5
Primary completed	20.9	1.1	34.7	1.3
Secondary/High school completed	17.7	0.9	30.7	1.2
College completed or above	13.5	1.3	32.5	2.6
**Employment**				
Currently in paid employment	17.4	0.7	26.1	1.0
Not working for pay	34.7	1.0	44.6	0.9
**Household economic status**				
Lowest quintile	34.4	1.3	49.3	1.4
Second quintile	27.6	1.4	41.9	1.3
Middle quintile	24.5	1.3	44.2	1.4
Forth quintile	21.2	1.1	34.3	1.7
Highest quintile	12.8	0.9	29.5	1.5
**Urban-rural residence**				
Rural area	26.9	0.9	42.8	1.2
Urban area	20.0	0.8	37.3	0.9

*All numbers are percentages.

Pooled analysis of 57 countries, World Health Survey, 2002-2004.

Table 2 shows the adjusted effects of the social determinants on disability resulting from the logistic regression models for males and females. There is a positive and significant association between age and disability. However, marital status has no significant effect once the other variables are controlled for. Lower levels of education are significantly associated with disability both among men and women. Likewise, the likelihood of being disabled is significantly higher among people who do not participate in the labor force, although this effect is stronger for men than for women. Also economic status is significantly related to disability, with increased disability prevalence associated with lower economic status, especially for men. In contrast, area of residence is not significantly associated with disability for either men or women once the effects of the other factors have been controlled for.

**Table 2:** Adjusted associations between disability and determinants in men and women aged 50 and older

		**Men**			**Women**		
		Odds ratio*	**95% CI**		Odds ratio*	**95% CI**	
**Age** (Reference category: 50–54 years)	55-59 years	**1.07**	0.88	1.30	**1.10**	0.92	1.31
	60-64 years	**1.22**	0.99	1.51	**1.70**	1.42	2.02
	65-69 years	**1.98**	1.60	2.45	**1.81**	1.52	2.16
	70-74 years	**1.95**	1.53	2.50	**2.61**	2.14	3.18
	75-79 years	**2.77**	2.12	3.62	**2.86**	2.31	3.54
	80+ years	**3.10**	2.34	4.12	**5.38**	4.28	6.77
**Marital status** (Reference category: Married/cohabiting)	Never married	**0.92**	0.64	1.32	**1.00**	0.80	1.26
	Divorced/separated/widow	**1.04**	0.70	1.53	**1.22**	0.96	1.54
**Education** (Reference category: College completed or above)	No education	**2.10**	1.55	2.86	**2.07**	1.53	2.78
	Incomplete primary	**2.24**	1.64	3.06	**2.00**	1.51	2.64
	Primary completed	**1.43**	1.07	1.90	**1.38**	1.07	1.79
	Secondary/High school completed	**1.30**	0.99	1.70	**0.99**	0.78	1.25
**Employment** (Reference category: Currently in paid employment)		**2.20**	1.89	2.57	**1.44**	1.26	1.66
**Household economic status** (Reference category: Highest quintile)	Lowest quintile	**2.56**	2.01	3.26	**1.56**	1.26	1.92
	Second quintile	**1.85**	1.46	2.34	**1.28**	1.05	1.57
	Middle quintile	**1.73**	1.36	2.19	**1.54**	1.26	1.88
	Forth quintile	**1.60**	1.29	1.98	**1.05**	0.86	1.29
**Urban-rural residence** (Reference category: Rural area)		**0.95**	0.81	1.11	**1.08**	0.93	1.25
		N = 28568			N = 35070		
		Pseudo R-squared 0.149			Pseudo R-squared 0.143		

*Odds ratios also adjusted for country of residence.

Pooled analysis of 57 countries, World Health Survey, 2002-2004.

The decomposition in Table 3 shows how the social determinants influence the difference in disability between older men and women. Approximately 45% of the inequality between men and women in this population can be attributed to differences in the *distribution* of socio-demographic factors. This is the so-called “explained” part of the inequality, meaning that this results from differences in the characteristics between older men and women. Of this “explained” inequality, 81% of the contribution comes from social determinants including employment (49%), education (15%), marital status (12%) and household economic status (4%). The remaining 19% of the “explained” inequality is attributed to differences in the distribution of age (10%) and country of residence (10%).

**Table 3:** Decomposition of difference in disability between men and women aged 50 and older

	**Estimate**	**SE**	
**Gender difference in disability prevalence**	**16.4**	**0.9**	
	**Absolute contribution**	**95% CI**	
**Explained**	**7.4**	**6.2**	**8.6**
Age	0.7	0.4	0.9
Marital status	0.9	0.4	1.4
Education	1.1	0.8	1.5
Household economic status	0.3	0.1	0.4
Employment	3.6	2.9	4.3
Urban-rural residence	0.0	−0.1	0.1
Country of residence	0.7	0.1	1.2
**Unexplained**	**9.0**	**7.2**	**10.8**
Age	−0.9	−1.9	0.0
Marital status	0.1	−2.3	2.6
Education	−0.2	−1.1	0.7
Household economic status	0.0	−0.1	0.1
Employment	−0.4	−0.7	−0.1
Urban-rural residence	−0.1	−0.2	0.1
Country of residence	0.8	−0.8	2.4
Constant	9.6	6.6	12.6

*All numbers are percentage points. Pooled analysis of 57 countries, World Health Survey, 2002–2004.

Approximately 55% of the sex difference in disability prevalence results from differences in the *effects* of the determinants on disability (Table 3). This includes factors in the model (age, country of residence, social determinants) as well as others not in the model. This is the “unexplained” part of the inequality in disability between men and women. The differential effects of age and country of residence contributed most to the “unexplained” part, although these effects were not statistically significant. With the exception of employment, the differential effects of the social determinants included in the model (i.e. education, marital status, urban–rural residence and household economic status) were smaller and not statistically significant.

## Discussion

### Prevalence of disability in men and women aged 50 and older

This analysis of household survey data from 57 countries demonstrates inequality in disability prevalence between men and women aged 50 and over. Prevalence of disability is higher among women than among men across all age groups. Prevalence of disability among women aged 50 to 54 years is higher than that among men aged 60 to 64 years. Other studies reported in the literature show that older aged women are more likely than men to become disabled and remain disabled, thus having longer duration of disability, particularly at very old ages [11,13,38-40].

In populations aged 50 and older, women fare worse in their health than men with regard to the social determinants. Specifically, women who completed their education to college level or above have higher disability than men who have no formal education. Women in the highest wealth quintile have higher disability than men in the second lowest wealth quintile. These results are also consistent with the literature showing that the health of older women in many parts of the world is worse for unemployment, lack of education and lower income [3,10,14-17,40,41].

### Multivariate analysis of social factors associated with disability in men and women aged 50 and older

The multivariate analysis for males and females shows that, after adjusting for marital status, education, employment, household economic status and area of residence, age is significantly positively associated with increasing disability, with a stronger gradient across age groups for older women. Compared to those with college education or above, men and women with no education or incomplete primary education are twice as likely to be disabled. Studies have examined associations between “disability” (measured in relation to difficulty performing common daily activities), education [14] and other socioeconomic factors [15,16]. Here we use a measure of disability based on the current ICF definition. This conceptualization captures people who are experiencing difficulties in executing tasks or actions on a continuum of functioning, regardless of whether this is due to chronic conditions such as heart disease or arthritis, or physical impairments such as blindness or paralysis.

Employment remains a major factor associated with the health of people aged 50 and older, and this is particularly important for older men [14]. We would expect stronger associations between unemployment and disability at the younger end of this age range (e.g. 50 to 64 years) [42] although these patterns are likely to vary between countries. The associations between employment and household economic status and disability are stronger for older men than older women, possibly reflecting differences in gender roles. A study of educational and income inequalities in morbidity among the elderly (60 and over) in eleven European countries showed inequalities in all age groups and countries and for both sexes [42].

### Decomposition of difference in disability between men and women aged 50 and older

The decomposition showed that approximately 45% of the inequality in disability between men and women aged 50 and over is attributed to the distribution of determinants between the sexes. Employment, education, marital status and household economic status, together and individually, made a significant contribution, and age and country were also major contributors to the explained component.

Employment was the largest single contributor to the “explained” component, this being due to the fact that a higher proportion of men than women were in paid jobs (63.2% vs. 24.2%). Analysis of WHS responses to a question in which people stated reasons for not working for pay showed that only 6.4% gave “ill health” as a reason. The main reasons given by women for not having a paid job were associated with being homemakers or caring for the family followed by being retired. Being retired was the single main reason for men.

Education also contributed to the “explained” component resulting from the fact that more women than men had no education (41.6% vs. 28.8%). The contribution of marital status to the “explained component” is due to the fact that a much higher proportion of women than men were divorced, widowed or separated (40.0% vs. 10.6%). For details see Additional file 3: Table S3.

We hypothesize that country specific social factors that we have not measured here (e.g. differences in occupational opportunities between men and women [43], differential access to social protection mechanisms, religious beliefs, family arrangements and cultural norms [18,44,45], also contribute to our measured inequality in disability among older men and women [9]. Our “country” variable is possibly a proxy for these factors although more research is needed to better understand these patterns.

More than half – approximately 55% – of the inequality in disability between these older men and women is due to the differential effects of the determinants we investigated as well as factors not included in our model. Employment made a statistically significant contribution to the “unexplained” component, because being unemployed is associated with higher odds of disability for men – 2.20 (95% CI: 1.89 to 2.57) – than for women – 1.44 (95% CI: 1.26 to 1.66). Biological (genetic, physiological and hormonal) differences between males and females determine male-specific responses to morbidity and co-morbidity.

### Strengths and limitations

Our research has several strengths. The study is the first to use a multi-country dataset to decompose the determinants of the inequality in disability among older men and women. The use of a large WHS dataset (57 countries) which is based on a consistent set of measures, ensures comparability in measuring disability prevalence and inequalities between men and women. Importantly, the decomposition has allowed us to explore social factors that compromise the health of older men and women. Research such as this is needed so that evidence-based policy responses can be developed [46].

Much of the literature on gender differences in disability in older populations focuses only on limitations in ADL and IADL [47]. The measurement of disability among adults aged 50 and older, was based on a parsimonious set of eight health domains (vision, mobility, self-care, cognition, interpersonal activities, pain and discomfort, sleep and energy, and affect) in order to give a more complete picture of disability. While the “unexplained” component of the inequality suggests that there are factors that contribute to the inequality that were either not assessed in the WHS or were not included in the present analysis, this is a strength rather than a limitation. Unlike other studies of inequalities in disability between men and women, we have used a decomposition method to identify the “explained” and “unexplained” components of the inequality. Breaking down the inequality in this way provides a platform for further research to better understand policy relevant factors that contribute to the inequality.

However this study has some limitations. Firstly the analysis is based on self-reported data, and so incurs the possibility of report bias whereby response is influenced by people's understanding of questions, their experiences, expectations, and culture. There are also differences in the way in which men and women describe their health. Women are more likely to report poorer functioning and worse overall health than men [48]. Future studies should include physical assessment of functioning in multiple domains to minimize report bias and calibrate self-reports. Secondly, the countries included here are not necessarily representative of all countries in the world, or of groups of countries defined by income or geographical region. Thirdly, the position that older women have in society in each of these countries is likely to vary from country to country and we were not able to explain how this occurred. Cultural and societal factors that place women in a subordinate position to men underlie and contribute to gender inequalities in health. It is possible that differences in disability result from interactions between sex (biological) gender (social), race, ethnicity, and other social and behavioral factors but we were unable to identify these influences here [9].

## Conclusions

Global ageing has a major influence on disability trends and national populations are ageing at unprecedented rates [3]. The important demographic and epidemiological shifts that are occurring internationally are being accompanied by increasing feminization of older adult populations due to differences in life expectancies between men and women. These trends are occurring in both developed and developing countries and they require policy actions that respond to the needs of disabled older adult populations [3]. Underpinning this policy imperative is the need for data and methodologies that can identify how social, biological and other factors separately contribute to the health decrements facing men and women as they age. While attention is often paid to health inequalities in younger populations, this study highlights the need for action to address social structures and institutional practices that impact unfairly on the health of older men and women.

## Competing interests

The authors declare that they have no competing interests.

## Authors’ contributions

AH designed the study and undertook the statistical analysis. JSW undertook the literature review and drafted the manuscript with input from AH and SC. BJ, AP, PK and AO read the draft and provided input and critical comment. All co-authors read and approved the final draft.

## Support

This paper uses data from WHO's World Health Surveys (WHS). The analysis for this paper was supported by the US National Institute on Aging through Interagency Agreements (OGHA 04034785; YA1323-08-CN-0020; Y1-AG-1005-01) and through a research grant (R01-AG034479). Additional support was provided by CBM International.

## Supplementary Material

Additional file 1 **Table S1.** Study sample size, by country. Pooled analysis of 57 countries, World Health Survey, 2002–2004. Click here for file

Additional file 2 **Table S2.** Questions on Health Domains. World Health Survey, 2002–2004. Click here for file

Additional file 3 **Table S3.** Distribution of determinants in men and women aged 50 and older. Pooled analysis of 57 countries, World Health Survey, 2002–2004. Click here for file
