# Supplementary material for: Social determinants of sex differences in disability among older adults: a multi-country decomposition analysis using the World Health Survey
Source: Int J Equity Health. 2012 Sep 8;11:52. doi: 10.1186/1475-9276-11-52 (PMC3463479; doi:10.1186/1475-9276-11-52)
Supplement: Additional file 1 — Table S1. Study sample size, by country. Pooled analysis of 57 countries, World Health Survey, 2002–2004. [file 1475-9276-11-52-S1.docx]

**Additional file 1: Table S1. Study sample size, by country. Pooled analysis of 57 countries, World Health Survey, 2002-2004**

| **Country** | **Men** | **Women** | **Combined** |
| --- | --- | --- | --- |
| Bangladesh | 609 | 578 | 1,187 |
| Bosnia and Herzegovina | 159 | 276 | 435 |
| Brazil | 640 | 783 | 1,423 |
| Burkina Faso | 480 | 416 | 896 |
| Chad | 437 | 473 | 910 |
| China | 690 | 698 | 1,388 |
| Comoros | 158 | 71 | 229 |
| Congo | 153 | 185 | 338 |
| Cote d'Ivoire | 276 | 191 | 467 |
| Croatia | 207 | 332 | 539 |
| Czech Republic | 179 | 242 | 421 |
| Dominican Republic | 647 | 676 | 1,323 |
| Ecuador | 536 | 565 | 1,101 |
| Estonia | 183 | 322 | 505 |
| Ethiopia | 560 | 485 | 1,045 |
| Finland | 260 | 324 | 584 |
| France | 44 | 29 | 73 |
| Georgia | 475 | 745 | 1,220 |
| Ghana | 458 | 574 | 1,032 |
| Hungary | 71 | 77 | 148 |
| India | 1,188 | 1,185 | 2,373 |
| Ireland | 140 | 136 | 276 |
| Israel | 102 | 177 | 279 |
| Kazakhstan | 396 | 703 | 1,099 |
| Kenya | 388 | 533 | 921 |
| Lao People's Democratic Republic | 350 | 378 | 728 |
| Latvia | 144 | 323 | 467 |
| Luxembourg | 124 | 131 | 255 |
| Malawi | 435 | 634 | 1,069 |
| Malaysia | 713 | 792 | 1,505 |
| Mali | 475 | 272 | 747 |
| Mauritania | 313 | 455 | 768 |
| Mauritius | 456 | 583 | 1,039 |
| Mexico | 4,718 | 6,146 | 10,864 |
| Morocco | 582 | 440 | 1,022 |
| Myanmar | 658 | 944 | 1,602 |
| Namibia | 306 | 479 | 785 |
| Nepal | 943 | 929 | 1,872 |
| Pakistan | 728 | 519 | 1,247 |
| Paraguay | 627 | 685 | 1,312 |
| Philippines | 966 | 1,232 | 2,198 |
| Portugal | 175 | 336 | 511 |
| Russian Federation | 698 | 1,545 | 2,243 |
| Senegal | 217 | 147 | 364 |
| Slovakia | 97 | 230 | 327 |
| South Africa | 193 | 234 | 427 |
| Spain | 1,385 | 2,003 | 3,388 |
| Sri Lanka | 756 | 801 | 1,557 |
| Swaziland | 190 | 210 | 400 |
| Sweden | 201 | 306 | 507 |
| Tunisia | 592 | 735 | 1,327 |
| United Arab Emirates | 242 | 158 | 400 |
| Ukraine | 343 | 706 | 1,049 |
| Uruguay | 534 | 624 | 1,158 |
| Viet Nam | 334 | 416 | 750 |
| Zambia | 299 | 393 | 692 |
| Zimbabwe | 338 | 508 | 846 |
|  |  |  |  |
| **Total** | **28,568** | **35,070** | **63,638** |

**Additional file 2 Table 2 Questions on Health Domains. World Health Survey, 2002-2004**

| **Self-reported assessment scale:**  **1.** None **2.** Mild **3.** Moderate **4.** Severe **5.** Extreme/Cannot Do | |
| --- | --- |
| **Mobility** | |
| Q2010 | Overall in the last 30 days, how much difficulty did you have with moving around? |
| Q2011 | In the last 30 days, how much difficulty did you have in vigorous activities, such as running 3 km (or equivalent) or cycling? |
| **Self Care** | |
| Q2020 | Overall in the last 30 days, how much difficulty did you have with selfcare, such as washing or dressing yourself? |
| Q2021 | In the last 30 days, how much difficulty did you have in taking care of and  maintaining your general appearance (e.g. grooming, looking neat and tidy etc.) |
| **Pain and Discomfort** | |
| Q2030 | Overall in the last 30 days, how much of bodily aches or pains did you have? |
| Q2031 | In the last 30 days, how much bodily discomfort did you have? |
| **Cognition** | |
| Q2050 | Overall in the last 30 days, how much difficulty did you have with  concentrating or remembering things? |
| Q2051 | In the last 30 days, how much difficulty did you have in learning a new task (for example, learning how to get to a new place, learning a new game, learning a new recipe etc.)? |
| **Interpersonal Activities** | |
| Q2060 | Overall in the last 30 days, how much difficulty did you have with personal relationship or participation in the community? |
| Q2061 | In the last 30 days, how much difficulty did you have in dealing with conflicts and tensions with others? |
| **Vision** |  |
| *Q2070 | Do you wear glasses or contact lenses? |
| Q2071 | In the last 30 days, how much difficulty did you have in seeing and recognizing a person you know across the road (i.e. from a distance of about 20 meters)? |
| Q2072 | In the last 30 days, how much difficulty did you have in seeing and recognizing an object at arm’s length or in reading? |
| **Sleep and Energy** | |
| Q2080 | Overall in the last 30 days, how much of a problem did you have with sleeping, such as falling asleep, waking up frequently during the night or waking up too early in the morning? |
| Q2081 | In the last 30 days, how much of a problem did you have due to not feeling rested and refreshed during the day (e.g. feeling tired, not having energy)? |
| **Affect** |  |
| Q2090 | Overall in the last 30 days, how much of a problem did you have with feeling sad, low or depressed? |
| Q2091 | Overall in the last 30 days, how much of a problem did you have with  worry or anxiety? |

*If respondent says YES to this question, preface questions 2071 and 2072 with "Please answer the following questions taking into account your glasses or contact lenses".

**Additional file 3 Table 3 Distribution of determinants in men and women aged 50 and older. Pooled analysis of 57 countries, World Health Survey, 2002-2004**

|  | **Men** | |  | **Women** | |
| --- | --- | --- | --- | --- | --- |
|  | **Sample Count** | **Weighted Proportion (%)** |  | **Sample Count** | **Weighted Proportion (%)** |
|  |  |  |  |  |  |
| **Overall** | 28568 |  |  | 35070 |  |
|  |  |  |  |  |  |
| **Age*** |  |  |  |  |  |
| 50-54 years | 7205 | 28.5 |  | 8902 | 27.2 |
| 55-59 years | 5235 | 21.0 |  | 6266 | 18.5 |
| 60-64 years | 4877 | 17.1 |  | 5932 | 17.2 |
| 65-69 years | 4023 | 12.7 |  | 4750 | 13.1 |
| 70-74 years | 3342 | 10.6 |  | 4207 | 11.1 |
| 75-79 years | 2022 | 5.9 |  | 2553 | 6.6 |
| 80+ years | 1864 | 4.2 |  | 2460 | 6.3 |
|  |  |  |  |  |  |
| **Marital status*** |  |  |  |  |  |
| Married/cohabiting | 22932 | 86.8 |  | 17294 | 56.3 |
| Never married | 1266 | 2.6 |  | 1922 | 3.7 |
| Divorced/separated/widowed | 4370 | 10.6 |  | 15854 | 40.0 |
|  |  |  |  |  |  |
| **Education*** |  |  |  |  |  |
| No education | 7103 | 28.8 |  | 10803 | 41.6 |
| Incomplete primary | 4456 | 14.0 |  | 5068 | 13.4 |
| Primary completed | 6075 | 19.2 |  | 6841 | 14.9 |
| Secondary/High school completed | 8703 | 27.8 |  | 10052 | 22.5 |
| College completed or above | 2231 | 10.2 |  | 2306 | 7.6 |
|  |  |  |  |  |  |
| **Employment*** |  |  |  |  |  |
| Currently in paid employment | 11624 | 63.2 |  | 25578 | 24.2 |
| Not working for pay | 16944 | 36.8 |  | 9492 | 75.8 |
|  |  |  |  |  |  |
| **Household economic status*** |  |  |  |  |  |
| Lowest quintile | 6172 | 17.3 |  | 8738 | 21.0 |
| Second quintile | 6083 | 20.7 |  | 7484 | 20.6 |
| Middle quintile | 5621 | 21.1 |  | 6789 | 19.7 |
| Forth quintile | 5450 | 20.2 |  | 6380 | 20.7 |
| Highest quintile | 5242 | 20.7 |  | 5679 | 18.0 |
|  |  |  |  |  |  |
| **Urban-rural residence*** |  |  |  |  |  |
| Rural area | 14514 | 55.0 |  | 16353 | 51.0 |
| Urban area | 14054 | 45.0 |  | 18717 | 49.0 |
|  |  |  |  |  |  |

*p value <0.001
