# Supplementary material for: Social determinants of sex differences in disability among older adults: a multi-country decomposition analysis using the World Health Survey
Source: Int J Equity Health. 2012 Sep 8;11:52. doi: 10.1186/1475-9276-11-52 (PMC3463479; doi:10.1186/1475-9276-11-52)
Supplement: Additional file 2 — Table S2. Questions on Health Domains. World Health Survey, 2002–2004. [file 1475-9276-11-52-S2.doc]

**Additional file 2: Table S2.** Questions on Health Domains. World Health Survey, 2002-2004

| **Self-reported assessment scale:**  **1.** None **2.** Mild **3.** Moderate **4.** Severe **5.** Extreme/Cannot Do | |
| --- | --- |
| **Mobility** | |
| Q2010 | Overall in the last 30 days, how much difficulty did you have with moving around? |
| Q2011 | In the last 30 days, how much difficulty did you have in vigorous activities, such as running 3 km (or equivalent) or cycling? |
| **Self Care** | |
| Q2020 | Overall in the last 30 days, how much difficulty did you have with selfcare, such as washing or dressing yourself? |
| Q2021 | In the last 30 days, how much difficulty did you have in taking care of and  maintaining your general appearance (e.g. grooming, looking neat and tidy etc.) |
| **Pain and Discomfort** | |
| Q2030 | Overall in the last 30 days, how much of bodily aches or pains did you have? |
| Q2031 | In the last 30 days, how much bodily discomfort did you have? |
| **Cognition** | |
| Q2050 | Overall in the last 30 days, how much difficulty did you have with  concentrating or remembering things? |
| Q2051 | In the last 30 days, how much difficulty did you have in learning a new task (for example, learning how to get to a new place, learning a new game, learning a new recipe etc.)? |
| **Interpersonal Activities** | |
| Q2060 | Overall in the last 30 days, how much difficulty did you have with personal relationship or participation in the community? |
| Q2061 | In the last 30 days, how much difficulty did you have in dealing with conflicts and tensions with others? |
| **Vision** |  |
| *Q2070 | Do you wear glasses or contact lenses? |
| Q2071 | In the last 30 days, how much difficulty did you have in seeing and recognizing a person you know across the road (i.e. from a distance of about 20 meters)? |
| Q2072 | In the last 30 days, how much difficulty did you have in seeing and recognizing an object at arm’s length or in reading? |
| **Sleep and Energy** | |
| Q2080 | Overall in the last 30 days, how much of a problem did you have with sleeping, such as falling asleep, waking up frequently during the night or waking up too early in the morning? |
| Q2081 | In the last 30 days, how much of a problem did you have due to not feeling rested and refreshed during the day (e.g. feeling tired, not having energy)? |
| **Affect** |  |
| Q2090 | Overall in the last 30 days, how much of a problem did you have with feeling sad, low or depressed? |
| Q2091 | Overall in the last 30 days, how much of a problem did you have with  worry or anxiety? |

*If respondent says YES to this question, preface questions 2071 and 2072 with "Please answer the following questions taking into account your glasses or contact lenses".
