# Supplementary material for: Social determinants of sex differences in disability among older adults: a multi-country decomposition analysis using the World Health Survey
Source: Int J Equity Health. 2012 Sep 8;11:52. doi: 10.1186/1475-9276-11-52 (PMC3463479; doi:10.1186/1475-9276-11-52)
Supplement: Additional file 3 — Table S3. Distribution of determinants in men and women aged 50 and older. Pooled analysis of 57 countries, World Health Survey, 2002–2004. [file 1475-9276-11-52-S3.doc]

**Additional file 3: Table S3.** Distribution of social determinants by sex, World Health Survey, 2002-2004

|  | **Men** | |  | **Women** | |
| --- | --- | --- | --- | --- | --- |
|  | **Sample Count** | **Weighted Proportion (%)** |  | **Sample Count** | **Weighted Proportion (%)** |
|  |  |  |  |  |  |
| **Overall** | 28568 |  |  | 35070 |  |
|  |  |  |  |  |  |
| **Age** |  |  |  |  |  |
| 50-54 years | 7205 | 28.5 |  | 8902 | 27.2 |
| 55-59 years | 5235 | 21.0 |  | 6266 | 18.5 |
| 60-64 years | 4877 | 17.1 |  | 5932 | 17.2 |
| 65-69 years | 4023 | 12.7 |  | 4750 | 13.1 |
| 70-74 years | 3342 | 10.6 |  | 4207 | 11.1 |
| 75-79 years | 2022 | 5.9 |  | 2553 | 6.6 |
| 80+ years | 1864 | 4.2 |  | 2460 | 6.3 |
|  |  |  |  |  |  |
| **Marital status** |  |  |  |  |  |
| Married/cohabiting | 22932 | 86.8 |  | 17294 | 56.3 |
| Never married | 1266 | 2.6 |  | 1922 | 3.7 |
| Divorced/separated/widowed | 4370 | 10.6 |  | 15854 | 40.0 |
|  |  |  |  |  |  |
| **Education** |  |  |  |  |  |
| No education | 7103 | 28.8 |  | 10803 | 41.6 |
| Incomplete primary education | 4456 | 14.0 |  | 5068 | 13.4 |
| Primary completed | 6075 | 19.2 |  | 6841 | 14.9 |
| Secondary/High school completed | 8703 | 27.8 |  | 10052 | 22.5 |
| College completed or above | 2231 | 10.2 |  | 2306 | 7.6 |
|  |  |  |  |  |  |
| **Employment** |  |  |  |  |  |
| Currently in paid employed | 11624 | 63.2 |  | 25578 | 24.2 |
| Not working for pay | 16944 | 36.8 |  | 9492 | 75.8 |
|  |  |  |  |  |  |
| **Household economic status** |  |  |  |  |  |
| Lowest quintile | 6172 | 17.3 |  | 8738 | 21.0 |
| Second quintile | 6083 | 20.7 |  | 7484 | 20.6 |
| Middle quintile | 5621 | 21.1 |  | 6789 | 19.7 |
| Forth quintile | 5450 | 20.2 |  | 6380 | 20.7 |
| Highest quintile | 5242 | 20.7 |  | 5679 | 18.0 |
|  |  |  |  |  |  |
| **Urban-rural residence** |  |  |  |  |  |
| Rural area | 14514 | 55.0 |  | 16353 | 51.0 |
| Urban area | 14054 | 45.0 |  | 18717 | 49.0 |
|  |  |  |  |  |  |

*p value <0.0001
